# Arterial stiffness tested by pulse wave velocity and augmentation index for cardiovascular risk stratification in antiphospholipid syndrome

**DOI:** 10.1093/rheumatology/kead267

**Published:** 2023-06-09

**Authors:** Gerasimos Evangelatos, George Konstantonis, Nikolaos Tentolouris, Petros P Sfikakis, Maria G Tektonidou

**Affiliations:** First Department of Propaedeutic Internal Medicine, Medical School, National and Kapodistrian University of Athens, Athens, Greece; Joint Academic Rheumatology Program, Medical School, National and Kapodistrian University of Athens, Athens, Greece; First Department of Propaedeutic Internal Medicine, Medical School, National and Kapodistrian University of Athens, Athens, Greece; First Department of Propaedeutic Internal Medicine, Medical School, National and Kapodistrian University of Athens, Athens, Greece; First Department of Propaedeutic Internal Medicine, Medical School, National and Kapodistrian University of Athens, Athens, Greece; Joint Academic Rheumatology Program, Medical School, National and Kapodistrian University of Athens, Athens, Greece; First Department of Propaedeutic Internal Medicine, Medical School, National and Kapodistrian University of Athens, Athens, Greece; Joint Academic Rheumatology Program, Medical School, National and Kapodistrian University of Athens, Athens, Greece

**Keywords:** APS, diabetes mellitus, cardiovascular risk, arterial stiffness, augmentation index

## Abstract

**Objectives:**

Cardiovascular disease is a major cause of morbidity and mortality in Antiphospholipid syndrome (APS). Arterial stiffness (ArS) has emerged as a predictor of future cardiovascular events in the general population. We aimed to assess ArS in patients with thrombotic APS versus diabetes mellitus (DM) and healthy controls (HC) and identify predictors of increased ArS in APS.

**Methods:**

ArS was evaluated by carotid–femoral pulse wave velocity (cfPWV) and augmentation index normalized to 75 beats/min (AIx@75) using the SphygmoCor device. Participants also underwent carotid/femoral ultrasound for atherosclerotic plaque detection. We used linear regression to compare ArS measures among groups and assess ArS determinants in the APS group.

**Results:**

We included 110 patients with APS (70.9% female, mean age 45.4 years), 110 DM patients and 110 HC, all age/sex matched. After adjustment for age, sex, cardiovascular risk factors and plaque presence, APS patients exhibited similar cfPWV [β = −0.142 (95% CI −0.514, 0.230), *p* = 0.454] but increased AIx@75 [β = 4.525 (95% CI 1.372, 7.677), *p* = 0.005] compared with HC and lower cfPWV (*p* < 0.001) but similar AIx@75 (*p* = 0.193) versus DM patients. In the APS group, cfPWV was independently associated with age [β = 0.056 (95% CI 0.034, 0.078), *p* < 0.001], mean arterial pressure (MAP) [β = 0.070 (95% CI 0.043, 0.097), *p* < 0.001], atherosclerotic femoral plaques [β = 0.732 (95% CI 0.053, 1.411), *p* = 0.035] and anti-β2-glycoprotein I IgM positivity [β = 0.696 (95% CI 0.201, 1.191), *p* = 0.006]. AIx@75 was associated with age [β = 0.334 (95% CI 0.117, 0.551), *p* = 0.003], female sex [β = 7.447 (95% CI 2.312, 12.581), *p* = 0.005] and MAP [β = 0.425 (95% CI 0.187, 0.663), *p* = 0.001].

**Conclusion:**

APS patients exhibit elevated AIx@75 *vs* HC and similar to DM patients, indicating enhanced arterial stiffening in APS. Given its prognostic value, ArS evaluation may help to improve cardiovascular risk stratification in APS.

Rheumatology key messages

- Augmentation index is increased in antiphospholipid syndrome (APS), similar to diabetes mellitus.
- Age, sex, mean arterial pressure, femoral plaques and anti-β2-glycoprotein I are associated with arterial stiffness in APS.
- Arterial stiffness evaluation can help to improve cardiovascular risk stratification in APS.

## Introduction

Antiphospholipid syndrome (APS) is a rare autoimmune disorder with increased morbidity and mortality that is mainly attributed to cardiovascular disease (CVD) events such as myocardial infarction and stroke [1]. A high burden of subclinical atherosclerosis, a predictor of CVD events, has also been demonstrated in APS [2, 3]. aPL-mediated thromboinflammation and atherothrombosis are emerging pathogenetic mechanisms of CVD in APS [4]. Traditional cardiovascular risk factors (CVRFs) are similarly or more prevalent in APS than in other high CVD risk disorders, such as diabetes mellitus (DM) and RA, but inadequately controlled in clinical practice [5, 6]. The recent EULAR recommendations for CVD risk management in rheumatic and musculoskeletal disorders (RMDs), including systemic lupus erythematosus (SLE) and APS [7], reported that the clinical risk scores underestimate CVD risk in RMDs and underlined the need to improve CVD risk stratification in these patients.

Accumulating data indicate arterial stiffness (ArS) as an independent predictor of future cardiovascular events and CVD-related mortality in the general population, beyond the effect of traditional CVRFs [8–10]. Given its prognostic value, evaluation of ArS may have an additive role in CVD risk stratification by recognizing high CVD risk individuals not detected by the clinical CVD risk scores [11]. Among RMDs, the majority of studies examined ArS in patients with inflammatory arthritis and SLE, detecting a high prevalence of ArS and associations with both traditional CVRFs and disease-related factors, such as disease duration and activity [12, 13]. Evidence has shown that chronic inflammation leads to endothelial dysfunction and structural changes in the vascular wall that can promote arterial stiffening [14]. Only sporadic studies have examined ArS in patients with APS [15–18], with their quality often being limited by small samples, mixed patient groups or exclusion of specific patient phenotypes, or the lack of multivariate analyses.

Herein we aimed to assess ArS in patients with thrombotic APS using validated markers, namely carotid–femoral pulse wave velocity (cfPWV) and augmentation index (AIx) [8, 9, 11], compared with healthy controls (HC) and patients with DM, a high CVD risk disorder with increased ArS [19]. In addition, we sought to identify predictors of ArS in APS.

## Methods

### Study design and population

All patients with thrombotic APS, either primary APS (PAPS) or secondary to SLE (SLE-APS), who were followed up in the Rheumatology Unit of the First Department of Propaedeutic and Internal Medicine at Laiko Hospital were assessed for eligibility for inclusion in this cross-sectional study. APS patients fulfilled the revised Sapporo classification criteria for definite APS [20], while SLE-APS patients also met the classification criteria for SLE [21]. Thrombotic events were confirmed by appropriate imaging studies or histopathological findings [20]. Individuals with a known history of atherosclerotic CVD events, chronic kidney disease (CKD) stage 5, isolated obstetric APS, active infection or malignancy were excluded. APS patients with concomitant DM were also excluded.

All included patients with APS were matched 1:1 for age and sex with DM patients and HC. Patients with DM (either type 1 or type 2) were followed up in the Diabetes Center of our department, while HC were recruited by our Cardiovascular Research Laboratory using community-based methods. Based on the available literature [18], it was calculated that a sample size of 93 individuals per group was required for 80% probability of demonstrating a difference of 0.7 m/sec in PWV between comparison groups (SD1 = 1.6, SD2 = 1.8) with a 5% significance level (two-tailed test).

The study was conducted according to the Declaration of Helsinki and was approved by our Hospital’s Institutional Review Board (Laiko Hospital Scientific Council; IRB 1041). All patients provided written informed consent.

### Clinical and laboratory recorded parameters

Demographics (age, sex, ethnicity/race) and the following clinical data were recorded for all participants: disease duration (for APS and DM patients), mean peripheral (brachial) arterial pressure (MAP), arterial hypertension, dyslipidaemia, smoking status, family history of premature CVD, exercise level (in min/week), BMI, CKD stage 3 or 4 and concomitant medications (statins, antihypertensives, antiplatelets). Antihypertensives were further reported by class: renin–angiotensin–aldosterone system (RAAS) inhibitors (angiotensin-converting enzyme inhibitors or angiotensin receptor blockers), calcium channel blockers (CCBs), diuretics and β-blockers. Laboratory data included serum creatinine, total cholesterol, high-density lipoprotein (HDL), low-density lipoprotein (LDL) and triglycerides.

Specifically for APS patients, disease-related parameters were additionally recorded: APS type (PAPS or SLE-APS), type of thrombotic events (venous or arterial), recurrent thrombotic events, history of obstetric events, aPLs (lupus anticoagulant [LA], IgG and IgM isotypes of anti-cardiolipin [aCL] and anti-β2-glycoprotein I [anti-β2GPI] antibodies), double or triple aPL positivity, adjusted global APS score (aGAPSS) and aGAPSS-CVD [22] and treatments (corticosteroids, hydroxychloroquine, immunosuppressives, anticoagulants). Extended blood and urine tests were performed the day of the vascular examination, including immunological tests such as aPLs, anti-double-stranded DNA (anti-dsDNA) antibodies and C3 and C4 levels. aCL and anti-β2GPI were measured using standardized ELISA (ImmunoWELL cardiolipin antibody, GenBio, San Diego, CA, USA for aCL; Imtec-ELISA, Human Gesellschaft für Biochemica und Diagnostica, Wiesbaden, Germany for anti-β2GPI) and LA was detected according to the guidelines of the Scientific Subcommittee of the International Society on Thrombosis and Haemostasis [23]. For patients with SLE-APS, anti-dsDNA antibodies were quantified using radioimmunoassay (RIA) and C3 and C4 levels using nephelometry. The SLEDAI 2000 (SLEDAI-2K) and SLICC-ACR Damage Index (SDI) were calculated to assess disease activity and disease damage, respectively.

### ArS and subclinical atherosclerosis evaluation

ArS was non-invasively assessed by measuring cfPWV and the pulse wave reflections by AIx using the SphygmoCor device (ATCOR, Sydney, NSW, Australia). The time interval between the arrival of the pulse wave at the common carotid artery and the common femoral artery was quantified. cfPWV was calculated as the carotid-to-femoral distance (as defined by subtracting the carotid measurement site to the sternal notch distance from the sternal notch to the femoral measurement site) divided by the pulse transmission time [24]. cfPWV was measured as m/s and the mean value of two consecutive measurements was recorded. In case the two measurements differed by at least 0.5 m/s, a third measurement was performed and the median value of all three measurements was recorded [24]. After acquiring 20 sequential stable radial waveforms through pulse wave analysis, an average radial and corresponding aortic pressure waveform could be generated in order to estimate pulse pressure and augmentation pressure. AIx was determined as the augmentation pressure:pulse pressure ratio and was expressed as a percentage (%). To minimize the influence of heart rate on the AIx [25], obtained AIx values were normalized for a heart rate of 75 beats/min (AΙx@75).

At the same vascular examination visit, the presence of atherosclerotic plaques was examined by vascular ultrasound in both far and near walls at eight arterial sites (right and left common carotid arteries, carotid bulbs, internal carotid arteries and common femoral arteries), according to the Mannheim Carotid Intima-Media Thickness (IMT) and Plaque Consensus [26]. A local thickening of the IMT of >1.5 mm or a local increase of the IMT >50% or >0.5 mm compared with the surrounding vessel wall was defined as atherosclerotic plaque. A high-resolution ultrasound (Vivid 7 Pro, GE Heathcare, Chicago, IL, USA) with a 14-MHz multifrequency linear probe was used.

All participants were asked to avoid food, tobacco, alcohol and caffeine consumption and vasoactive medication use for 12 hours prior to the ultrasound examination. All measurements were carried out between 08:00 and 10:00 in a quiet, dimly lit room, with controlled stable temperature (approximately 23°C), after at least 10 minutes of rest in a supine position. All assessments were performed by the same experienced operator (G.K.), who was blinded to the participant’s clinical data. Intra-observer variability was assessed by a between-measures coefficient of variation and was calculated as 0.03 (3%) indicating good method performance.

### Statistical analysis

Continuous variables were presented as either mean (standard deviation [SD])) or median (interquartile range [IQR]), depending on their distribution, and categorical variables as frequencies and percentages. The Kolmogorov–Smirnov test was performed to assess the normality of sample distribution. Comparisons between continuous variables were performed with Student’s *t*-test and analysis of variance or Mann–Whitney and Kruskal–Wallis test, depending on the normality of distribution. We also performed post hoc pairwise multiple comparison using Bonferroni correction and Dunn’s test as appropriate. Comparisons of categorical variables were performed by the chi-squared test.

We used linear regression models to compare cfPWV and AIx@75 among the APS, DM and HC groups. In multivariate models we included variables that were found to be significant (*p* < 0.10) in the univariate analyses (Supplementary Tables S2 and S3, available at *Rheumatology* online); if multiple CVRFs were found significant, we included MAP instead of hypertension in the multivariate model to avoid overlap, and LDL instead of total cholesterol, HDL or triglycerides, since LDL has been recommended as the primary target in lipid management guidelines [27]. Except from a first extensive model (model 1) for each outcome (cfPWV and AIx@75), two additional multivariate analyses (models 2 and 3) were performed with a declining number of included confounders. In models 2 and 3 we used the variable ‘number of traditional CVRFs’ instead of each CVRF separately, while ‘antihypertensives’ and ‘statins’ were not included in model 3 in order to further reduce the number of variables, as they were partially overlapping with ‘number of traditional CVRFs’.

Multiple linear regression analyses were applied within the APS group to assess determinants of increased ArS in APS patients. Clinical and laboratory parameters significantly associated (*p* < 0.10) with either cfPWV or AIx@75 in the univariate analysis (Supplementary Tables S4 and S5, available at *Rheumatology* online) were included in the multivariate models.

To reduce the number of included variables, we used the ‘number of traditional CVRFs’ as an independent variable rather than including each CVRF separately, keeping age and MAP in all models, since they are recognized as the main determinants of PWV and AIx in the general population [28, 29].

*p-*values in multivariate models were considered statistically significant if they were <0.05. Analyses were performed using Stata, version 13.0 (StataCorp, College Station, TX, USA).

## Results

### Study group characteristics

**Figure 1.**
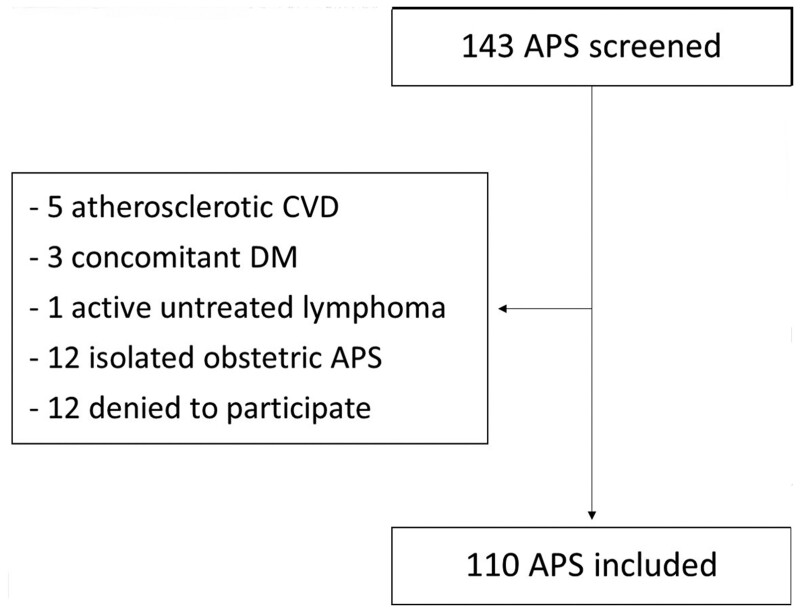
Study flowchart

We screened 143 patients from our APS reference centre cohort, 33 of whom were excluded based on our exclusion criteria (Fig. 1). In total, 330 participants were included in the study (Table 1): 110 APS patients [70.9% female, mean age 45.4 years (SD 12.2), 61.8% PAPS, 38.2% SLE-APS, 47.2% triple aPL positive) and 110 age- and sex-matched DM patients and 110 age- and sex-matched HC. The characteristics of PAPS and SLE-APS patients are presented in Table 2. In SLE-APS patients, the median SLEDAI-2K was 2 (IQR 0–4); only 7 of 42 SLE-APS patients (16.7%) had active disease (SLEDAI-2K >4) at the time of vascular examination and all had a SLEDAI-2K ≤7. The median SDI was 1 (IQR 0–2). Among DM patients, 43.6% had type 2 DM, median haemoglobin A1c was 7.3% (IQR 6.8–8.3), while 41.8% were on oral antidiabetic drugs and 70.0% on insulin treatment. APS and DM patients had higher BMI, smoking pack-years and anti-platelets use compared with HC (Table 1). Similar results were seen after applying Bonferroni correction and Dunn’s test (Supplementary Table S1, available at *Rheumatology* online). In addition, as we have previously described [2, 3], patients with APS had a higher prevalence of atherosclerotic plaques in the carotid and/or femoral arteries than HC (33.6% *vs* 14.5%; *p* = 0.001) and comparable to that in DM patients (33.6% *vs* 31.8%; *p* = 0.774).

**Table 1.** Characteristics of study subgroups

Characteristics	APS	DM	HC	*p-*value^a^	*p-*value^b^	*p-*value^c^	*p-*value^d^
Age, years, mean (s.d.)	45.4 (12.2)	45.6 (12.4)	45.4 (12.3)	0.873	0.969	0.904	0.986
Female, *n* (%)	78 (70.9)	78 (70.9)	78 (70.9)	1.000	1.000	1.000	1.000
Disease duration, years, median (IQR)	6.5 (2–16)	12.5 (5–21.5)	–	**0.0001**	–	–	–
Family history of premature CVD, *n* (%)	15 (13.6)	15 (13.6)	14 (12.7)	1.000	0.842	0.842	0.974
Current smoker, *n* (%)	41 (37.3)	41 (37.3)	36 (32.7)	1.000	0.480	0.480	0.719
Smoking, pack-years, mean (s.d.)	10.5 (14.4)	14.6 (21.7)	7.2 (11.5)	0.805	**0.024**	**0.025**	**0.004**
Arterial hypertension, *n* (%)	32 (29.1)	37 (33.6)	31 (28.2)	0.467	0.881	0.381	0.641
Blood pressure (brachial), mmHg, mean (s.d.)	90.5 (9.7)	92.1 (10.8)	93.0 (11.1)	0.393	0.076	0.527	0.206
Dyslipidaemia, *n* (%)	27 (24.8)	38 (36.9)	24 (23.3)	0.056	0.802	**0.033**	0.058
Total cholesterol, mg/dl, mean (s.d.)	186.4 (37.1)	196.6 (36.6)	204.0 (36.2)	**0.046**	**<0.001**	0.146	**0.002**
LDL, mg/dl, mean (s.d.)	107.8 (33.3)	117.9 (34.4)	122.9 (32.4)	**0.025**	**0.001**	0.345	**0.004**
HDL, mg/dl, mean (s.d.)	55.9 (16.4)	54.8 (14.9)	61.1 (17.7)	0.663	**0.030**	**0.008**	**0.018**
Triglycerides, mg/dl, median (IQR)^b^	90 (70–133)	90 (66–142)	84 (63–123)	0.815	0.320	0.548	0.625
CKD (stage 3–4), *n* (%)	7 (6.4)	6 (5.9)	3 (2.9)	0.898	0.234	0.292	0.465
BMI, kg/m^2^, mean (s.d.)	27.5 (5.1)	28.5 (6.0)	26.0 (4.5)	0.385	**0.027**	**0.002**	**0.002**
Exercise level, min/week, median (IQR)	0 (0–180)	90 (0–300)	110 (0–180)	0.065	**0.044**	0.943	0.108
Traditional CVRFs, *n* (%)							
0–1	66 (60)	52 (51.0)	63 (61.2)	0.187	0.862	0.142	0.270
≥2	44 (40)	50 (49.0)	40 (38.8)				
Anti-hypertensives, *n* (%)	30 (27.3)	37 (33.6)	19 (17.3)	0.764	0.075	**0.038**	0.090
RAAS inhibitor	19 (17.3)	32 (29.1)	13 (11.8)	**0.038**	0.251	**0.001**	**0.004**
CCB	5 (4.5)	14 (12.7)	7 (6.4)	**0.031**	0.553	0.108	0.061
Diuretic	6 (5.5)	12 (10.9)	5 (4.5)	0.140	0.757	0.077	0.134
β-blocker	12 (10.9)	7 (6.4)	6 (5.5)	0.230	0.140	0.775	0.261
Statin, *n* (%)	18 (16.4)	32 (29.1)	10 (9.1)	**0.024**	0.106	**<0.001**	**0.001**
Anti-platelets, *n* (%)	40 (36.4)	10 (9.1)	2 (1.8)	**<0.001**	**<0.001**	**0.018**	**<0.001**

Values in bold are statistically significant.

APS: antiphospholipid syndrome; DM: diabetes mellitus; HC: healthy controls; CVD: cardiovascular disease; LDL: low density lipoprotein; HDL: high density lipoprotein; CKD: chronic kidney disease; BMI: Body mass index; CVRF: cardiovascular risk factors; RAASi: renin-angiotensinaldosterone system inhibitors (angiotensin-converting enzyme inhibitors or angiotensin receptor blockers); CCBs: calcium channel blockers.

a APS *vs* DM.

b APS *vs* HC.

c DM *vs* HC.

d Between the three groups.

**Table 2.** Characteristics of patients with PAPS and SLE-APS

Characteristics	PAPS (*n* = 68)	SLE-APS (*n* = 42)	*p-*value
Age, years, mean (s.d.)	45.8 (13.2)	44.6 (10.6)	0.624
Female, *n* (%)	41 (60.3)	37 (88.1)	**0.002**
Disease duration, years, median (IQR)	6 (3–12)	9.5 (1.5–19)	0.329
Family history of premature CVD, *n* (%)	11 (16.2)	4 (9.5)	0.323
Current smoker, *n* (%)	29 (42.7)	12 (28.6)	0.138
Smoking, pack-years, mean (s.d.)	11.9 (15.9)	8.3 (11.6)	0.202
Arterial hypertension, *n* (%)	20 (29.4)	12 (28.6)	0.925
Blood pressure (brachial), mmHg, mean (s.d.)	91.0 (9.3)	89.7 (10.3)	0.483
Dyslipidaemia, *n* (%)	19 (28.4)	8 (19.1)	0.273
Total cholesterol, mg/dl, mean (s.d.)	188.6 (33.7)	185.9 (42.4)	0.920
LDL, mg/dl, mean (s.d.)	106.9 (31.8)	109.2 (35.8)	0.730
HDL, mg/dl, mean (s.d.)	56.1 (16.1)	55.7 (7.1)	0.898
Triglycerides, mg/dl, median (IQR)	90 (70–133)	88 (74–131)	0.937
CKD (stage 3–4), *n* (%)	4 (5.9)	3 (7.1)	0.792
BMI, kg/m^2^, mean (s.d.)	27.9 (4.5)	26.8 (5.8)	0.273
Exercise level, min/week, median (IQR)	0 (0–128)	90 (0–210)	**0.051**
Traditional CVRFs, *n* (%)			
0–1	35 (51.5)	31 (73.8)	**0.020**
≥2	33 (48.5)	11 (26.2)	
Anti-hypertensives, *n* (%)	16 (23.5)	14 (33.3)	0.262
RAAS inhibitor	11 (16.2)	8 (19.5)	0.699
CCB	4 (5.9)	1 (2.4)	0.392
Diuretic	5 (7.4)	1 (2.4)	0.265
β-blocker	6 (8.8)	6 (14.3)	0.372
Statin, *n* (%)	15 (22.1)	3 (7.1)	**0.040**

aCL IgG positivity, *n* (%)	41 (60.3)	32 (76.2)	0.086
aCL IgM positivity, *n* (%)	33 (48.5)	22 (52.4)	0.695
anti-β2GPI IgG positivity, *n* (%)	33 (48.5)	17 (40.5)	0.410
anti-β2GPI IgM positivity, *n* (%)	29 (42.7)	16 (38.1)	0.637
LA positivity, *n* (%) (*n* = 67/*n* = 41)	53 (79.1)	31 (75.6)	0.672
aPL positivity, *n* (%) (*n* = 67/*n* = 41)			
Single positivity	17 (25.4)	9 (22.0)	
Double positivity	18 (26.9)	13 (31.7)	0.843
Triple positivity	32 (47.8)	19 (46.3)	
Recurrent thromboses, *n* (%)	34 (50.0)	15 (35.7)	0.143
Arterial thromboses, *n* (%)	39 (57.4)	16 (38.1)	**0.050**
Venous thromboses, *n* (%)	39 (57.4)	29 (69.1)	0.220
Obstetric APS^a^, *n* (%)	17 (41.5)	12 (32.4)	0.410
Corticosteroids (current), *n* (%)	5 (7.4)	22 (52.4)	**<0.001**
Cumulative corticosteroids dose, mg [PAPS, median (IQR); SLE-APS, mean (s.d.)]	0 (0–207)	14 808 (21 097)	**<0.001**
HCQ (current), *n* (%)	18 (26.5)	28 (66.7)	**<0.001**
Cumulative HCQ use, months, mean (s.d.)	14.5 (35.5)	78.1 (84.3)	**<0.001**
Immunosuppressives (current), *n* (%)	5 (7.4)	20 (47.6)	**<0.001**
Anticoagulants, *n* (%)	62 (91.2)	32 (76.2)	**0.030**
Antiplatelets, *n* (%)	22 (32.4)	18 (42.9)	0.266
aGAPSS, mean (s.d.)	9.9 (4.5)	9.3 (4.5)	0.467
aGAPSS-CVD, mean (s.d.)	11.2 (4.5)	10.6 (4.8)	0.486
SLEDAI-2K, median (IQR)	N/A	2 (0–4)	N/A
SLEDAI-2K >4 (active disease), *n* (%)	N/A	7 (16.7)	N/A
SDI, median (IQR)	N/A	1 (0–2)	N/A

Values in bold are statistically significant.

APS: antiphospholipid syndrome; PAPS: Primary APS; SLE: Systemic Lupus Erythematosus; CVD: Cardiovascular disease; LDL: low-density lipoprotein; HDL: high-density lipoprotein; CKD: chronic kidney disease; BMI: body mass index; CVRFs: cardiovascular risk factors; RAASi: renin-angiotensin-aldosterone system inhibitors (angiotensin-converting enzyme inhibitors or angiotensin receptor blockers); CCBs: calcium channel blockers; aCL: anticardiolipin antibodies; anti-β2GPI: anti-beta2-glycoprotein I antibodies; LA: Lupus Anticoagulant; aPL: antiphospholipid; HCQ: hydroxychloroquine; aGAPSS: adjusted Global Anti-Phospholipid Syndrome Score; SLEDAI-2K: Systemic Lupus Erythematosus Disease Activity Index 2000; SDI: Systemic Lupus International Collaborating Clinics-American College of Rheumatology Damage Index.

a Calculated for the group of female patients (*n* = 41 for PAPS, *n* = 37 for SLE-APS).

### ArS comparison between groups

The median cfPWV was 7.05 m/sec (IQR 6.25–8.05) in patients with APS *vs* 7.9 (6.9–9.4) and 7.3 (6.7–8.1) in patients with DM and HC, respectively. The median AIx@75 was 27% (IQR 17–36) in APS patients and 25.5 (19.5–35) and 26 (11–34) in the DM and HC groups, respectively. In the subgroups of participants without any concomitant traditional CVRF (16 APS patients, 23 DM patients and 35 HC), the median cfPWV was 6.33 m/sec (IQR 5.99–6.74), 6.90 (6.20–7.55) and 7.05 (6.50–7.75), respectively, and the median AIx@75 was 17.8% (IQR 10.9–27.3), 18.0 (−3.0–27.0) and 16.0 (3.5–31.0), respectively.

After controlling for parameters found to be statistically significant in univariate analysis (Supplementary Table S2, available at *Rheumatology* online), namely age, smoking (pack-years), MAP, LDL, BMI, anti-hypertensive and statin use, CKD and plaque presence (either in the carotid or femoral arteries), cfPWV levels did not differ between APS patients and HC [β = −0.142 (95% CI −0.514, 0.230), *p* = 0.454] (model 1 for cfPWV, Table 3), but were higher in DM patients compared with APS patients [β = −0.863 (95% CI −1.223, −0.500), *p* < 0.001]. Similar results were obtained from two additional models including fewer variables (models 2 and 3 for cfPWV, Table 3).

**Table 3.** Multivariate analysis of cfPWV and AIx@75 between APS, DM and HC.

	Β coefficient	95% CI	*p-*value
cfPWV			
Model 1^a^			
APS *vs* HC	−0.142	−0.514, 0.230	0.454
APS *vs* DM	**−0.863**	**−1.223, −0.500**	**<0.001**
Model 2^b^			
APS *vs* HC	−0.142	−0.498, 0.214	0.434
APS *vs* DM	**−0.909**	**−1.263, −0.555**	**<0.001**
Model 3^c^			
APS *vs* HC	−0.133	−0.489, 0.223	0.464
APS *vs* DM	**−0.904**	**−1.254, −0.554**	**<0.001**
AΙx@75			
Model 1^d^			
APS *vs* HC	**4.525**	**1.372, 7.677**	**0.005**
APS *vs* DM	2.046	−1.040, 5.132	0.193
Model 2^e^			
APS *vs* HC	**4.164**	**1.259, 7.069**	**0.005**
APS *vs* DM	1.750	−1.165, 4.665	0.238
Model 3^f^			
APS *vs* HC	**4.124**	**1.222, 7.027**	**0.006**
APS *vs* DM	2.017	−0.857, 4.891	0.168

Values in bold are statistically significant.

cfPWV: carotid-femoral pulse wave velocity; AIx@75: augmentation index normalized for heart rate of 75 bpm; CI: confidence interval; APS: antiphospholipid syndrome; DM: diabetes mellitus; HC: healthy controls; MAP: mean arterial pressure (brachial); LDL: low density lipoprotein; CKD: chronic kidney disease (stages III-IV); BMI: body mass index.

a Adjusted for age, MAP (brachial), smoking (pack-years), LDL, BMI, anti-hypertensives and statins use, CKD (stages 3–4) and plaque presence.

b Adjusted for age, MAP (brachial), number of traditional CVRFs, anti-hypertensives and statins use, CKD (stages 3–4) and plaque presence.

c Adjusted for age, MAP (brachial), number of traditional CVRFs, CKD (stages 3–4) and plaque presence.

d Adjusted for age, sex, MAP (brachial), smoking (pack-year), LDL levels, BMI, anti-hypertensives, statins and anti-platelets use, exercise, CKD (stages 3–4) and plaque presence.

e Adjusted for age, sex, MAP (brachial), number of traditional CVRFs, anti-hypertensives and statins use, exercise, CKD (stages 3–4) and plaque presence.

f Adjusted for age, sex, MAP (brachial), number of traditional CVRFs, exercise, CKD (stages 3–4) and plaque presence.

Increased AIx@75 values were observed in APS patients compared with HC [β = 4.525 (95% CI 1.372, 7.677), *p* = 0.005] (model 1 for AIx@75, Table 3), after adjusting for age, sex, smoking (pack-years), MAP, LDL, exercise, BMI, CKD, plaque presence and use of anti-hypertensives, statins and anti-platelets, based on univariate analysis (Supplementary Table S3, available at *Rheumatology* online). In the same model, the APS and DM groups exhibited comparable AIx@75 values [β = 2.046 (95% CI −1.040, 5.132), *p* = 0.193]. Additional analyses with fewer variables, revealed the same associations (models 2 and 3 for AIx@75, Table 3).

cfPWV and AIx@75 did not differ significantly between the two APS subgroups. The median cfPWV was 7.18 m/sec (IQR 6.23–8.20) in patients with PAPS and 6.68 m/sec (IQR 6.25–7.90) in SLE-APS patients (*p* = 0.285). The median AIx@75 was also comparable between the two subgroups: 26.5% (IQR 14.0–36.3) *vs* 28.0% (19.0–35.5) in PAPS and SLE-APS patients, respectively (*p* = 0.379).

### cfPWV and AIx@75 associations with disease-specific characteristics and traditional CVRFs in APS patients

In the multiple linear regression analysis within the APS subgroup, age, MAP and femoral plaque presence were independently associated with cfPWV levels (*p* < 0.001, *p* < 0.001 and *p* = 0.035, respectively) (Table 4). In the same model, a 0.696 m/s increase in cfPWV values was observed in patients with anti-β2GPI IgM positivity (95% CI 0.201, 1.191; *p* = 0.006) compared with anti-β2GPI IgM-negative APS patients. Regarding AIx@75, age, female sex and MAP were independent predictors of elevated AIx@75 levels (*p* = 0.003, *p* = 0.005, *p* = 0.001, respectively) (Table 4). No association was observed between cfPWV or AIx@75 and disease duration, APS type (PAPS or SLE-APS), type of thrombotic events (arterial or venous), recurrent events, double or triple aPL positivity, aGAPSS or aGAPSS-CVD, disease activity (SLEDAI-2K), disease damage (SDI) and disease-related medications (Table 4, Supplementary Tables S4 and S5, available at *Rheumatology* online).

**Table 4.** Multivariate linear regression models for parameters associated with cfPWV and AIx@75 within the APS group

Variables	Β coefficient	95% CI	*p-*value
cfPWV			
Age	**0.056**	**0.034, 0.078**	**<0.001**
MAP (brachial)	**0.070**	**0.043, 0.097**	**<0.001**
Number of traditional CVRF	−0.021	−0.296, 0.254	0.881
CKD (stages 3–4)	0.083	−0.969, 1.135	0.876
Femoral plaque presence	**0.732**	**0.053, 1.411**	**0.035**
anti-β2GPI IgM	**0.696**	**0.201, 1.191**	**0.006**
aCL IgG	−0.327	−0.856, 0.267	0.223
Venous events (yes *vs* no)	−0.013	−0.549, 0.523	0.962
AΙx@75			
Age	**0.334**	**0.117, 0.551**	**0.003**
Sex	**7.447**	**2.312, 12.581**	**0.005**
MAP (brachial)	**0.425**	**0.187, 0.663**	**0.001**
Number of traditional CVRF	0.666	−1.810, 3.142	0.595
Overall plaque presence	2.244	−3.319, 7.806	0.425
Disease duration	0.203	−0.097, 0.502	0.183
anti-β2GPI IgG	−2.072	−6.661, 2.517	0.373
Cumulative HCQ duration	0.008	−0.028, 0.044	0.657

Values in bold are statistically significant.

cfPWV: carotid-femoral pulse wave velocity; AIx@75: augmentation index normalized for heart rate of 75 bpm; CI: confidence interval; MAP: mean arterial pressure (brachial); CVRF: cardiovascular risk factors; CKD: chronic kidney disease (stages III-IV); anti-β2GPI: antibeta2-glycoprotein I antibodies; aCL: anti-cardiolipin antibodies; HCQ: hydroxychloroquine.

## Discussion

This is the first study, to our knowledge, examining both PWV and AIx values in patients with APS versus HC and the first that compared ArS values between patients with APS and age- and sex-matched patients with DM. We found higher AIx@75 values in APS patients than in HC and, importantly, values comparable to those for patients with DM, a prototype high CVD risk disorder.

PWV and AIx are surrogate measures of ArS [8, 11]. PWV is an established index of aortic stiffness while AIx incorporates both aortic stiffness and systemic vascular resistance, also providing information about wave reflection and arterial circulation [30, 31]. These two indexes are complementary but not interchangeable, and both predict future cardiovascular events and death in the general population and high CVD risk groups, independent of traditional CVRFs.

In APS, a previous study showed similar cfPWV values for 27 women with PAPS patients <55 years of age and age- and BMI-matched healthy women [15]. In another study, higher carotid–radial PWV levels were observed in 77 aPL-positive women (23 with obstetric PAPS, 38 with thrombotic PAPS and 16 asymptomatic aPL carriers) *vs* 77 age- and CVRF-matched healthy females (*p* = 0.04), without any significant differences among the three aPL-positive subgroups (*p* = 0.50) [18]. In multivariate analysis, adjustment for age, BMI, blood pressure, smoking and hypercholesterolaemia was performed, while we also included CKD and subclinical atherosclerosis as potential confounders, since both have been linked with increased ArS [9, 32]. Due to the heterogeneous population in this study and the different methodology used for PWV assessment (carotid–radial PWV), no direct comparison can be made with our results. A smaller study reported increased cfPWV in 22 PAPS patients with a history of arterial thrombosis *vs* 26 aPL-negative HC with comparable age, sex and CVRF prevalence; notably, patients with venous thrombotic events were excluded and multivariate analysis was not performed [16]. The only previous study that examined AIx in APS showed a significant association between AIx and carotid IMT and flow-mediated dilatation (a measure of endothelial function), but no comparison with HC was made [33]. We demonstrated for the first time that APS patients have elevated AIx@75 levels comparable to those in DM.

Age and MAP were the strongest independent predictors of both cfPWV and AIx@75 in our study, in accordance with the evidence in the general population [28, 29]. Aging leads to the replacement of elastic fibres from stiffer collagenous fibres in the aortic wall, resulting in increased aortic stiffness, which is also accelerated by chronic hypertension and vascular calcifications [34]. In a stiffened aorta, pulse waves travel faster to the peripheral arteries (increased PWV) and are reflected back to the aorta earlier [34]. As a result, systolic pulse pressure increases (increased AIx) and diastolic pulse pressure decreases [34]. The former increases the left ventricular afterload and provokes microvascular damage in low-impedance organs (mainly kidney and brain), while the latter can lead to diminished coronary artery perfusion and left ventricular ischaemia, dysfunction and hypertrophy [8, 34]. Concerning the association between antihypertensives and ArS, no difference was observed among various classes of anti-hypertensive drugs (e.g. RAAS inhibitors, CCBs, diuretics and β-blockers).

Female sex is another major determinant of AIx in the general population and high-risk groups, such as patients with hypertension [34, 35]. In our study, women had approximately 7.4 units higher AIx@75 compared with male APS patients. Although a possible explanation might be the shorter height in women [36], AIx remained higher in females after controlling for height [35].

In our study, only 16 of 110 APS patients and 35 of 110 HC were without any concomitant traditional CVRF. This is in agreement with previous studies, including two from national cohorts [5, 37], that showed a high prevalence of CVRFs in APS patients, higher than that in matched healthy individuals for the majority of CVRFs. It has been previously shown that PAPS patients without traditional CVRFs have endothelial function comparable to that of HC [38]. Although it would be interesting to compare ArS between APS patients and HC in the absence of traditional CVRFs, the very low number (*n* = 16) of this subgroup of APS patients precluded further statistical analysis.

Among disease-related features, we found an association between cfPWV and anti-β2GPI IgM positivity. Interestingly, Parra *et al.* [39] have also shown a significant association between AIx levels and positive anti-β2GPI IgM in SLE patients (*p* = 0.035). We also demonstrated that cfPWV was independently associated with femoral artery atherosclerosis. PWV has been associated with coronary, cerebral and carotid atherosclerosis and peripheral artery disease in the general population [9, 40], suggesting that arterial stiffening and atherosclerosis may share common pathogenic pathways [9]. Chronic inflammation and subsequent oxidative stress lead to endothelial dysfunction, vascular smooth muscle cell (VSMC) proliferation and changes in extracellular matrix composition [14, 41]. VSMCs can also convert into an osteoblastic phenotype, generating local mineralization and calcification deposits [42]. All these mechanisms constitute a vicious cycle of vascular remodelling, wall stiffening and atheroma formation. In APS, aPL-mediated vascular inflammation, oxidative stress and activation of monocytes, neutrophils and the complement system results in endothelial cell proliferation, fibrous intimal hyperplasia, VSMC dysregulation and a concurrent atherogenic process via oxidized LDL and β2GPI complexes [4, 43], which, in concert with traditional CVRF-mediated endothelial dysfunction, might lead to vascular stiffening.

Regarding the APS subtypes, no statistically significant difference was found in ArS markers between patients with PAPS and those with SLE-APS. In a previous study by Jurcut *et al.* [44], patients with SLE-APS exhibited increased PWV but similar AIx values compared with PAPS individuals. Methodological differences (PWV was measured locally in the right common carotid), the small sample size (10 PAPS and 12 SLE-APS patients) and the lack of multivariate analysis do not allow comparisons with our results. We did not observe any association between cfPWV or AIx@75 values and the use of corticosteroids (either current use or cumulative dose), hydroxychloroquine (current or cumulative use) or immunosuppressants. Also, no association was found between ArS (cfPWV or AIx@75) and disease activity (assessed by SLEDAI-2K) or disease damage (assessed by SDI) in the SLE-APS subgroup. However, it should be noted that only 16.7% of SLE-APS patients had active disease (SLEDAI > 4) at the time of vascular examination, and the median SDI was 1 (IQR 0–2).

The 2022 EULAR recommendations for CVD risk management in patients with RMDs, including SLE and APS [7], as well as the 2019 EULAR recommendations for the management of APS in adults [45], underlined the importance of risk stratification in these patients. PWV and AIx have been recognized as prognostic markers for future cardiovascular events, CVD-related mortality and all-cause mortality in the general population, independent of traditional CVRFs [10, 11]. Generic risk scores may underestimate CVD risk in systemic autoimmune diseases [7, 46, 47], where, in addition to traditional CVRFs, disease-related risk factors contribute to high CVD risk [7]. Patients of intermediate CVD risk as estimated by the classic CVD risk scores might be reclassified to a higher risk category after evaluation of ArS [11] and would benefit from early preventive therapeutic intervention [8]. Evidence has shown that improvement in AIx can reduce the incidence of cardiovascular events [11, 48], while PWV response following 1-year anti-hypertensive treatment has been associated with a 42% reduction in all-cause mortality [11]. It would also be of interest to examine the utility of ArS measurement in triple-positive aPL carriers without a documented thrombotic event, as triple aPL positivity is associated with an increased burden of other vascular ultrasound-detected features such as IMT and atherosclerotic plaques in APS [49].

There are several strengths in this study: this is the largest study of its kind in the APS literature considering the rarity of APS; it is the first study assessing possible associations between increased ArS and multiple traditional CVRFs, disease-related characteristics and subclinical atherosclerosis; and, importantly, all vascular examinations (for cfPWV, AIx@75 and carotid and femoral atherosclerosis) were conducted by the same experienced operator. Some limitations should also be recognized. Diabetic APS patients and patients with isolated obstetric APS were not included. This is because DM was a disease control group in the study, while obstetric APS pathophysiology may differ from thrombotic APS, as the former is not complicated by vascular damage [50, 51]. In addition, all patients included in the study were white Europeans, thus we can’t draw conclusions for patients of other race/ethnic backgrounds.

In conclusion, patients with APS exhibit similar cfPWV but increased AIx@75 compared with healthy individuals, in a comparable degree to that in DM, implying impaired vascular health. Age, sex, MAP and anti-β2GPI IgM positivity are independently associated with ArS, suggesting that both traditional CVRFs and disease-specific factors contribute to ArS in APS. The association of ArS with subclinical atherosclerosis further supports that ArS is part of the CVD burden in APS and might prove useful in CVD risk assessment by revealing high-risk individuals who would benefit from early interventions. Prospective studies are needed to further examine the utility of ArS markers in CVD risk stratification and management of APS patients.

## Supplementary Material

kead267_Supplementary_Data

## Data Availability

The data that support the findings of this study are available from the corresponding author upon reasonable request.
