# Supplementary material for: Arterial stiffness tested by pulse wave velocity and augmentation index for cardiovascular risk stratification in antiphospholipid syndrome
Source: Rheumatology (Oxford). 2023 Jun 9;63(4):1030–8. doi: 10.1093/rheumatology/kead267 (PMC10986810; doi:10.1093/rheumatology/kead267)
Supplement: kead267_Supplementary_Data [file kead267_supplementary_data.docx]

**Supplementary Material**

**Arterial stiffness tested by pulse wave velocity and augmentation index for cardiovascular risk stratification in antiphospholipid syndrome**

Gerasimos Evangelatos, George Konstantonis, Nikolaos Tentolouris, Petros P. Sfikakis, and Maria G. Tektonidou

**Supplementary Table S1.** Post-hoc pairwise multiple comparison of baseline characteristics of the three study groups

|  | **APS** | **DM** | **HC** | p-value^1^ | p-value^2^ | p-value^3^ | p-value^4^ |
| --- | --- | --- | --- | --- | --- | --- | --- |
| Age, years* | 45.4 ± 12.2 | 45.6 ± 12.4 | 45.4 ± 12.3 | 0.987 | 0.999 | 0.993 | 0.986 |
| Sex (female), n (%) | 78 (70.9) | 78 (70.9) | 78 (70.9) | 1.000 | 1.000 | 1.000 | 1.000 |
| Disease Duration, years† | 6.5 (2-16) | 12.5 (5-21.5) | - | **0.0001** | **-** | **-** | - |
| Family history of premature CVD, n (%) | 15 (13.6) | 15 (13.6) | 14 (12.7) | 1.000 | 0.842 | 0.842 | 0.974 |
| Smoking, current, n (%) | 41 (37.3) | 41 (37.3) | 36 (32.7) | 1.000 | 0.480 | 0.480 | 0.719 |
| Smoking, pack-years* | 10.5 ± 14.4 | 14.6 ± 21.7 | 7.2 ± 11.5 | 0.190 | 0.414 | **0.030** | **0.004** |
| Arterial hypertension, n (%) | 32 (29.1) | 37 (33.6) | 31 (28.2) | 0.467 | 0.881 | 0.381 | 0.641 |
| Mean blood pressure * (brachial), mmHg | 90.5 ± 9.7 | 92.1 ± 10.8 | 93.0 ± 11.1 | 0.809 | 0.237 | 0.999 | 0.206 |
| Dyslipidemia, n (%) | 27 (24.8) | 38 (36.9) | 24 (23.3) | 0.056 | 0.802 | **0.033** | 0.058 |
| Total cholesterol, mg/dL* | 186.4 ± 37.1 | 196.6 ± 36.6 | 204.0 ± 36.2 | 0.131 | **0.002** | 0.444 | **0.002** |
| LDL, mg/dL* | 107.8 ± 33.3 | 117.9 ± 34.4 | 122.9 ± 32.4 | 0.088 | **0.003** | 0.881 | **0.004** |
| HDL, mg/dL* | 55.9 ± 16.4 | 54.8 ± 14.9 | 61.1 ± 17.7 | 0.999 | 0.081 | **0.024** | **0.018** |
| Triglycerides, mg/dL† | 90 (70-133) | 90 (66-142) | 84 (63-123) | 0.388 | 0.171 | 0.256 | 0.625 |
| CKD (Stage III-IV), n (%) | 7 (6.4) | 6 (5.9) | 3 (2.9) | 0.898 | 0.234 | 0.292 | 0.465 |
| BMI, kg/m^2^* | 27.5 ± 5.1 | 28.5 ± 6.0 | 26.0 ± 4.5 | 0.402 | 0.111 | **0.001** | **0.002** |
| Exercise level, min/week† | 0 (0-180) | 90 (0-300) | 110 (0-180) | **0.028** | **0.027** | 0.494 | 0.108 |
| Number of traditional CVRFs 0-1, n (%) ≥2, n (%) | 66 (60)  44 (40) | 52 (51.0)  50 (49.0) | 63 (61.2)  40 (38.8) | 0.187 | 0.862 | 0.142 | 0.270 |
| Anti-hypertensives, n (%)  RAASi  CCBs  Diuretics  Beta-blockers | 30 (27.3)  19 (17.3)  5 (4.5)  6 (5.5)  12 (10.9) | 37 (33.6)  32 (29.1)  14 (12.7)  12 (10.9)  7 (6.4) | 19 (17.3)  13 (11.8)  7 (6.4)  5 (4.5)  6 (5.5) | 0.764  **0.038**  **0.031**  0.140  0.230 | 0.075  0.251  0.553  0.757  0.140 | **0.038**  **0.001**  0.108  0.077  0.775 | 0.090  **0.004**  0.061  0.134  0.261 |
| Statins, n (%) | 18 (16.4) | 32 (29.1) | 10 (9.1) | **0.024** | 0.106 | **<0.001** | **0.001** |
| Anti-platelets, n (%) | 40 (36.4) | 10 (9.1) | 2 (1.8) | **<0.001** | **<0.001** | **0.018** | **<0.001** |

APS: antiphospholipid syndrome, DM: diabetes mellitus, HC: healthy controls, CVD: cardiovascular disease, LDL: low density lipoprotein, HDL: high density lipoprotein, CKD: chronic kidney disease, BMI: Body mass index, CVRF: cardiovascular risk factors, RAASi: renin-angiotensin-aldosterone system inhibitors (angiotensin-converting enzyme inhibitors or angiotensin receptor blockers), CCBs: calcium channel blockers

*Mean (SD), †Median (IQR)
p-value^1^: APS vs DM, p-value^2^: APS vs HC, p-value^3^: DM vs HC**,** p-value^4^: between the 3 groups.
Values in bold are statistically significant.

**Supplementary Table S2.** Univariate analysis for cfPWV in the whole cohort

|  | **β coefficient** | **95% CI** | **p-value** |
| --- | --- | --- | --- |
| APS vs HC  APS vs DM | -0.217  **-0.996** | -0.677, 0.244  **-1.456, -0.535** | 0.355  **<0.001** |
| Age | **0.081** | **0.068, 0.094** | **<0.001** |
| Sex (female vs male) | 0.177 | -0.248, 0.603 | 0.413 |
| Family history of premature CVD | 0.077 | -0.492, 0.646 | 0.790 |
| Smoking, pack-years | **0.020** | **0.009, 0.032** | **<0.001** |
| Arterial hypertension | **1.905** | **1.538, 2.271** | **<0.001** |
| MAP | **0.090** | **0.074, 0.105** | **<0.001** |
| Dyslipidemia | **0.754** | **0.317, 1.190** | **0.001** |
| Total cholesterol | **0.005** | **0.0001, 0.011** | **0.046** |
| LDL | **0.008** | **0.003, 0.014** | **0.005** |
| HDL | **-0.020** | **-0.032, -0.008** | **0.001** |
| Triglycerides | **0.005** | **0.002, 0.008** | **0.001** |
| CKD (Stage III-IV) | **1.036** | **0.127, 1.944** | **0.026** |
| BMI | **0.091** | **0.056, 0.126** | **<0.001** |
| Exercise level | -0.0005 | -0.001, 0.0005 | 0.306 |
| Number of traditional CVRFs | **0.617** | **0.455, 0.778** | **<0.001** |
| Anti-hypertensives use  RAASi  CCBs  Diuretics  Beta blockers | **1.400**  **1.702**  **1.763**  **1.388**  0.202 | **0.977, 1.822**  **1.249, 2.155**  **1.071, 2.455**  **0.644, 2.133**  -0.528, 0.933 | **<0.001**  **<0.001**  **<0.001**  **<0.001**  0.587 |
| Statins use | **0.918** | **0.420, 1.403** | **<0.001** |
| Anti-platelets | -0.015 | -0.546, 0.515 | 0.955 |
| Overall plaques presence | **1.431** | **1.022, 1.840** | **<0.001** |
| Carotid plaques presence | **1.324** | **0.850, 1.798** | **<0.001** |
| Femoral plaques presence | **1.640** | **1.164, 2.116** | **<0.001** |

Values in bold are statistically significant.

cfPWV: carotid-femoral pulse wave velocity, CI: confidence interval, APS: antiphospholipid syndrome, DM: diabetes mellitus, HC: healthy controls, CVD: cardiovascular disease, MAP: mean arterial pressure, LDL: low density lipoprotein, HDL: high density lipoprotein, CKD: chronic kidney disease, BMI: Body mass index, CVRFs: cardiovascular risk factors, RAASi: renin-angiotensin-aldosterone system inhibitors (angiotensin-converting enzyme inhibitors or angiotensin receptor blockers), CCBs: calcium channel blockers.

**Supplementary Table S3.** Univariate analysis for AIx@75 in the whole cohort

|  | **β coefficient** | **95% CI** | **p-value** |
| --- | --- | --- | --- |
| APS vs HC  APS vs DM | 3.595  1.659 | -0.187, 7.378  -2.124, 5.442 | 0.062  0.389 |
| Age | **0.609** | **0.501, 0.716** | **<0.001** |
| Sex (females vs males) | **15.225** | **12.239, 18.211** | **<0.001** |
| Family history of premature CVD | 0.846 | -3.713, 5.406 | 0.715 |
| Smoking, pack-years | **0.157** | **0.066, 0.249** | **0.001** |
| Arterial hypertension | **9.005** | **5.776, 12.233** | **<0.001** |
| MAP | **0.415** | **0.275, 0.555** | **<0.001** |
| Dyslipidemia | **5.054** | **1.525, 8.584** | **0.005** |
| Total cholesterol | **0.078** | **0.035, 0.120** | **<0.001** |
| LDL | **0.078** | **0.031, 0.125** | **0.001** |
| HDL | -0.057 | -0.156, 0.041 | 0.253 |
| Triglycerides | **0.040** | **0.018, 0.063** | **0.001** |
| CKD (Stage III-IV) | **7.464** | **0.203, 14.724** | **0.044** |
| BMI | **0.451** | **0.163, 0.739** | **0.002** |
| Exercise level | **-0.012** | **-0.020, -0.005** | **0.002** |
| Number of traditional CVRFs | **3.852** | **2.505, 5.200** | **<0.001** |
| Anti-hypertensives use  RAASi  CCBs  Diuretics  Beta blockers | **6.307**  **5.561**  **6.332**  **6.900**  **6.671** | **2.771, 9.844**  **1.687, 9.435**  **0.619, 12.045**  **0.858, 12.942**  **0.858, 12.485** | **0.001**  **0.005**  **0.030**  **0.025**  **0.025** |
| Statins use | **5.202** | **1.222, 9.181** | **0.011** |
| Anti-platelets | **4.242** | **0.012, 8.472** | **0.049** |
| Overall plaques presence | **7.624** | **4.218, 11.031** | **<0.001** |
| Carotid plaques presence | **8.786** | **4.934, 12.638** | **<0.001** |
| Femoral plaques presence | **4.547** | **0.504, 8.589** | **0.028** |

Values in bold are statistically significant.

AIx@75: augmentation index normalized to 75 beats/minute, CI: confidence interval, APS: antiphospholipid syndrome, DM: diabetes mellitus, HC: healthy controls, CVD: cardiovascular disease, MAP: mean arterial pressure, LDL: low density lipoprotein, HDL: high density lipoprotein, CKD: chronic kidney disease, BMI: Body mass index, CVRFs: cardiovascular risk factors, RAASi: renin-angiotensin-aldosterone system inhibitors (angiotensin-converting enzyme inhibitors or angiotensin receptor blockers), CCBs: calcium channel blockers.

**Supplementary Table S4.** Univariate analysis for cfPWV within APS group

|  | **β coefficient** | **95% CI** | **p-value** |
| --- | --- | --- | --- |
| *Disease-specific characteristics* | | | |
| Disease Duration | 0.022 | -0.017, 0.062 | 0.269 |
| APS type (PAPS vs SLE-APS) | 0.444 | -0.218, 1.107 | 0.187 |
| aCL IgG positivity | -0.607 | -1.284, 0.070 | 0.078 |
| aCL IgM positivity | -0.016 | -0.665, 0.633 | 0.961 |
| anti-β2GPI IgG positivity | -0.470 | -1.116, 0.175 | 0.152 |
| anti-β2GPI IgM positivity | 0.616 | -0.034, 1.265 | 0.063 |
| LA positivity | -0.366 | -1.154, 0.421 | 0.358 |
| aPL positivity  double vs single  triple vs single | 0.429  0.005 | -0.478, 1.336  -0.817, 0.826 | 0.351  0.991 |
| Recurrent Thromboses | 0.412 | -0.237, 1.060 | 0.211 |
| Arterial thromboses | 0.586 | -0.053, 1.226 | 0.072 |
| Venous thromboses | **-0.701** | **-1.355, -0.047** | **0.036** |
| Obstetric APS* (yes vs no) | -0.088 | -0.757, 0.582 | 0.795 |
| Corticosteroids, current | 0.024 | -0.730, 0.778 | 0.951 |
| Cumulative corticosteroids dose | 0.001 | -0.001, 0.001 | 0.931 |
| HCQ, current | -0.339 | -0.994, 0.316 | 0.307 |
| Cumulative HCQ use | 0.001 | -0.005, 0.005 | 0.944 |
| Immunosuppressives, current | -0.579 | -1.369, 0.211 | 0.149 |
| aGAPSS | 0.023 | -0.051, 0.097 | 0.541 |
| aGAPSS-CVD | 0.018 | -0.055, 0.090 | 0.632 |
| SLEDAI-2K | 0.065 | -0.108, 0.238 | 0.454 |
| SDI | 0.057 | -0.301, 0.416 | 0.749 |
| *CVRFs* | | | |
| Age | **0.075** | **0.052, 0.097** | **<0.001** |
| Sex (female vs male) | -0.395 | -1.105, 0.316 | 0.273 |
| Family history of premature CVD | **-1.047** | **-1.971, -0.123** | **0.027** |
| Smoking, pack-years | **0.035** | **0.014, 0.057** | **0.002** |
| Arterial hypertension | **1.272** | **0.600, 1.944** | **<0.001** |
| MAP | **0.082** | **0.052, 0.112** | **<0.001** |
| Dyslipidemia | **1.068** | **0.337, 1.798** | **0.005** |
| Total cholesterol | 0.008 | -0.001, 0.017 | 0.070 |
| LDL | **0.011** | **0.002, 0.021** | **0.024** |
| HDL | -0.004 | -0.025, 0.016 | 0.666 |
| Triglycerides | 0.003 | -0.003, 0.009 | 0.315 |
| CKD (Stage III-IV) | **1.557** | **0.261, 2.852** | **0.019** |
| BMI | 0.031 | -0.033, 0.095 | 0.341 |
| Exercise level | -0.001 | -0.003, 0.001 | 0.338 |
| Traditional CVRF, number | **0.428** | **0.119, 0.737** | **0.007** |
| Anti-hypertensives use  RAASi  CCBs  Diuretics  Beta blockers | 0.500  0.820  0.575  1.027  -0.781 | -0.223, 1.222  -0.024, 1.664  -0.979, 2.129  -0.389, 2.442  -1.811, 0.249 | 0.173  0.057  0.465  0.153  0.136 |
| Statins use | **1.315** | **0.475, 2.156** | **0.002** |
| Anti-platelets | 0.377 | -0.294, 1.048 | 0.268 |
| Overall plaques presence | **1.216** | **0.569, 1.862** | **<0.001** |
| Carotid plaques presence | **0.777** | **0.038, 1.516** | **0.040** |
| Femoral plaques presence | **1.185** | **0.432, 1.937** | **0.002** |

Values in bold are statistically significant
*Within the group of women with APS

cfPWV: carotid-femoral pulse wave velocity, CI: confidence interval, PAPS: Primary APS, SLE: Systemic Lupus Erythematosus, aCL: anti-cardiolipin antibodies, anti-b2GPI: anti-beta2-glycoprotein I antibodies, LA: Lupus Anticoagulant, aPL: antiphospholipid, HCQ: hydroxychloroquine, aGAPSS: adjusted Global Anti-Phospholipid Syndrome Score, CVD: Cardiovascular disease, SLEDAI-2K: Systemic Lupus Erythematosus Disease Activity Index 2000; SDI: Systemic Lupus International Collaborating Clinics-American College of Rheumatology Damage Index, CVRFs: cardiovascular risk factors, MAP: mean arterial pressure, LDL: low density lipoprotein, HDL: high density lipoprotein, CKD: chronic kidney disease, BMI: Body mass index, RAASi: renin-angiotensin-aldosterone system inhibitors (angiotensin-converting enzyme inhibitors or angiotensin receptor blockers), CCBs: calcium channel blockers

**Supplementary Table S5.** Univariate analysis for AIx@75 within APS group

|  | **β coefficient** | **95% CI** | **p-value** |
| --- | --- | --- | --- |
| *Disease-specific characteristics* | | | |
| Disease Duration | **0.527** | **0.218, 0.837** | **0.001** |
| APS type (PAPS vs SLE-APS) | -1.509 | -6.993, 3.974 | 0.586 |
| aCL IgG positivity | -4.436 | -10.019, 1.147 | 0.118 |
| aCL IgM positivity | -1.909 | -7.232, 3.414 | 0.479 |
| anti-β2GPI IgG positivity | **-5.878** | **-11.116, -0.638** | **0.028** |
| anti-β2GPI IgM positivity | -2.032 | -7.446, 3.380 | 0.458 |
| LA positivity | -3.015 | -9.435, 3.405 | 0.354 |
| aPL positivity  double vs single  triple vs single | 3.068  -1.567 | -4.300, 10.437  -8.244, 5.110 | 0.411  0.643 |
| Recurrent Thromboses | 1.140 | -4.223, 6.503 | 0.674 |
| Arterial thromboses | 2.709 | -2.601, 8.020 | 0.314 |
| Venous thromboses | -1.683 | -7.165, 3.799 | 0.544 |
| Obstetric APS* (yes vs no) | -1.900 | -7.859, 4.058 | 0.527 |
| Corticosteroids, current | 3.037 | -3.135, 9.209 | 0.332 |
| Cumulative corticosteroids dose | 0.001 | -0.001, 0.001 | 0.186 |
| HCQ, current | -0.366 | -5.774, 5.042 | 0.894 |
| Cumulative HCQ use | 0.036 | -0.004, 0.076 | 0.076 |
| Immunosuppressives, current | 0.047 | -6.513, 6.607 | 0.989 |
| aGAPSS | 0.022 | -0.579, 0.624 | 0.942 |
| aGAPSS-CVD | 0.096 | -0.493, 0.684 | 0.748 |
| SLEDAI-2K | 0.673 | -1.184, 2.530 | 0.468 |
| SDI | 2.681 | -1.079, 6.442 | 0.157 |
| *CVRFs* | | | |
| Age | **0.565** | **0.374, 0.756** | **<0.001** |
| Sex (female vs male) | **9.601** | **4.020, 15.182** | **0.001** |
| Family history of premature CVD | -2.823 | -10.587, 4.923 | 0.471 |
| Smoking, pack-years | **0.184** | **0.001, 0.366** | **0.049** |
| Arterial hypertension | **8.049** | **2.380, 13.719** | **0.006** |
| MAP | **0.484** | **0.223, 0.746** | **<0.001** |
| Dyslipidemia | 2.704 | -3.503, 8.910 | 0.390 |
| Total cholesterol | 0.038 | -0.035, 0.110 | 0.307 |
| LDL | 0.045 | -0.036, 0.127 | 0.271 |
| HDL | 0.053 | -0.112, 0.219 | 0.524 |
| Triglycerides | 0.004 | -0.046, 0.054 | 0.876 |
| CKD (Stage III-IV) | 8.205 | -2.611, 19.021 | 0.136 |
| BMI | 0.001 | -0.530, 0.531 | 0.998 |
| Exercise level | -0.005 | -0.020, 0.010 | 0.528 |
| Traditional CVRF, number | **2.951** | **0.384, 5.518** | **0.025** |
| Anti-hypertensives use  RAASi  CCBs  Diuretics  Beta blockers | 3.696  4.125  5.086  6.968  1.782 | -2.253, 9.644  -2.889, 11.138  -7.685, 17.856  -4.704, 18.640  -6.768, 10.333 | 0.221  0.246  0.432  0.239  0.680 |
| Statins use | 6.216 | -0.897, 13.329 | 0.086 |
| Anti-platelets | 1.696 | -3.840, 7.233 | 0.545 |
| Overall plaques presence | **6.574** | **1.068, 12.079** | **0.020** |
| Carotid plaques presence | **6.620** | **0.551, 12.689** | **0.033** |
| Femoral plaques presence | 3.923 | -2.493, 10.339 | 0.228 |

Values in bold are statistically significant
*Within the group of women with APS

AIx@75: augmentation index normalized for heart rate of 75 bpm, CI: confidence interval, PAPS: Primary APS, SLE: Systemic Lupus Erythematosus, aCL: anti-cardiolipin antibodies, anti-b2GPI: anti-beta2-glycoprotein I antibodies, LA: Lupus Anticoagulant, aPL: antiphospholipid, HCQ: hydroxychloroquine, aGAPSS: adjusted Global Anti-Phospholipid Syndrome Score, CVD: Cardiovascular disease, SLEDAI-2K: Systemic Lupus Erythematosus Disease Activity Index 2000; SDI: Systemic Lupus International Collaborating Clinics-American College of Rheumatology Damage Index, CVRFs: cardiovascular risk factors, MAP: mean arterial pressure, LDL: low density lipoprotein, HDL: high density lipoprotein, CKD: chronic kidney disease, BMI: Body mass index, RAASi: renin-angiotensin-aldosterone system inhibitors (angiotensin-converting enzyme inhibitors or angiotensin receptor blockers), CCBs: calcium channel blockers.
